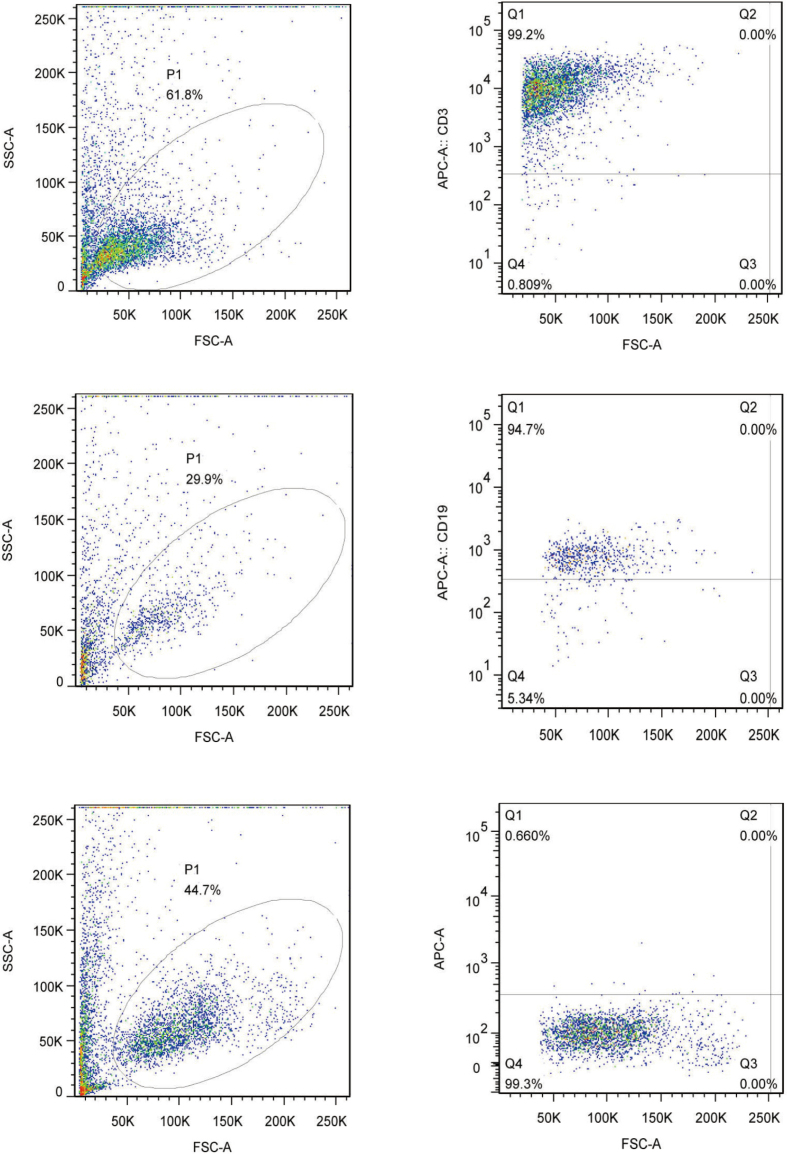# The link between ten-eleven translocation-2 (Tet2) related clonal hematopoiesis and sequential onset of two hematologic malignancies

**DOI:** 10.1016/j.gendis.2024.101270

**Published:** 2024-03-22

**Authors:** Jin Huang, Haolong Lin, Xia Mao, Min Xiao, Xiaoxi Zhou, Qilin Ao, Liang Huang

**Affiliations:** Department of Hematology, Tongji Hospital, Tongji Medical College, Huazhong University of Science and Technology, Wuhan, Hubei 430030, China; Institute of Pathology, Tongji Hospital, Tongji Medical College, Huazhong University of Science and Technology, Wuhan, Hubei 430030, China; Department of Pathology, School of Basic Medical Science, Tongji Medical College, Huazhong University of Science and Technology, Wuhan, Hubei 430030, China; Department of Hematology, Tongji Hospital, Tongji Medical College, Huazhong University of Science and Technology, Wuhan, Hubei 430030, China; State Key Laboratory of Experimental Hematology, National Clinical Research Center for Blood Diseases, Haihe Laboratory of Cell Ecosystem, Institute of Hematology & Blood Diseases Hospital, Chinese Academy of Medical Science & Peking Union Medical College, Tianjin 300020, China; Tianjin Institutes of Health Science, Tianjin 301600, China

Age-related clonal hematopoiesis is defined as the presence of somatic mutations of genes known to be recurrently mutated in hematologic malignancies in the peripheral blood of healthy individuals.^1^ When clonal hematopoiesis is associated with a mutation at a variant allele frequency of 0.02 or greater, it is termed “clonal hematopoiesis of indeterminate potential” (CHIP). Classical CHIP genes include epigenetic modifiers such as Dnmt3a, Tet2, and Asxl1, and DNA repair genes such as Tp53 and Ppm1d.^2^ Although CHIP-associated mutations are most frequent in myeloid neoplasm, some, such as those in Dnmt3a and Tet2, are recurrently observed in lymphoid malignancies as well. Tiacci et al described a case of sequential onset of angioimmunoblastic T-cell lymphoma possessing Tet2 mutations and acute myeloid leukemia within two years in a 45-year-old man.^3^ This suggested the potential roles of CHIP in the molecular pathogenesis of the sequential/simultaneous onset of two hematologic malignancies, also termed hematological composite malignancies (HCM). However, till now, the association of CHIP mutations with the clone evolution of HCM remains poorly clarified probably due to a low incidence.

For this purpose, we retrospectively analyzed ten patients with HCM in our center from 2015 to 2022 (Table S1). Ten patients were enrolled, all of whom were initially diagnosed with lymphoma (Table S1). Six cases (No. 5–10) were diagnosed with acute myeloid leukemia as a second hematologic malignancy during treatment, and a second lymphoma occurred in three patients (No. 2–4). One patient (No. 1) was diagnosed with diffuse large B-cell lymphoma combined with angioimmunoblastic T-cell lymphoma when first admitted to our center. The mean age of the patients was 49.2 years old ranging from 19 to 69 years, with seven males and three females. The median interval between the two malignancies was 25 months. In total, 70% of patients achieved complete remission for the first malignancy, and only 50% could achieve remission for the second neoplasms. By July 2023, six of these patients died, and the median survival time was 30.5 months. Other clinical characteristics and treatments are summarized in Table S1 and S2.

To investigate the molecular background of HCM and trace the clone origin, we performed a genetic analysis of paired samples (No. 1–9). The method of sequencing is provided in the supplement material. We found CHIP-associated gene mutations were highly prevalent in HCM patients (Fig. 1A). Tet2 gene mutations were most frequently detected (5 of 9 patients) in these patients, followed by Tp53 (3/9), Dnmt3a (2/9), Jak 2 (1/9), and Srsf2 (1/9). No mutations were detected in other CHIP-associated genes such as Asxl1, Bcorl1, Cbl, Gnas, Gnb1, Ppm1d, and Sf3b1 (see Table S1, 3 for details). Through high-throughput sequencing analysis, we found the two lineage tumors that shared the same genetic mutations in five patients (No. 1, 3, 5, 6, and 9; the details are shown in Fig. 1B–F), suggesting bidirectional evolution of clonal hematopoiesis. While for the other four patients, two different malignancies of the same individual exhibited completely divergent genetic background. Noteworthy, among the five patients sharing the same genetic origin, Tet2 gene mutations were exclusively detected in all the five patients, which indicated the two lineage malignant clones may originate from a common earlier Tet2-mutant cloning. In addition, the shared Tet2 mutations are almost truncating mutations resulting in a loss-of-function TET2 protein. To further illustrate the tumor origin and process of clonal evolution, flow cytometry-sorted bone marrow sub-populations from two patients (No. 1 and 9) were separately analyzed. Targeted next-generation sequencing of the lymphoid tissue, sorted B-lineage (CD19^+^) cells, and T-lineage cells (CD3^+^, in No. 1), or myeloid leukemia cells (CD34^+^, in No. 9) all identified the same Tet2 mutations (Fig. 1E, F; Fig. S1). All these results indicated the Tet2-CHIP mutation was probably acquired in the early hematopoietic stem/progenitor cell (HSC/HPC) stage, and then evolved to divergent second lymphoid or myeloid malignancies under certain exposure pressure or a second genetic hit.

**Figure 1:**
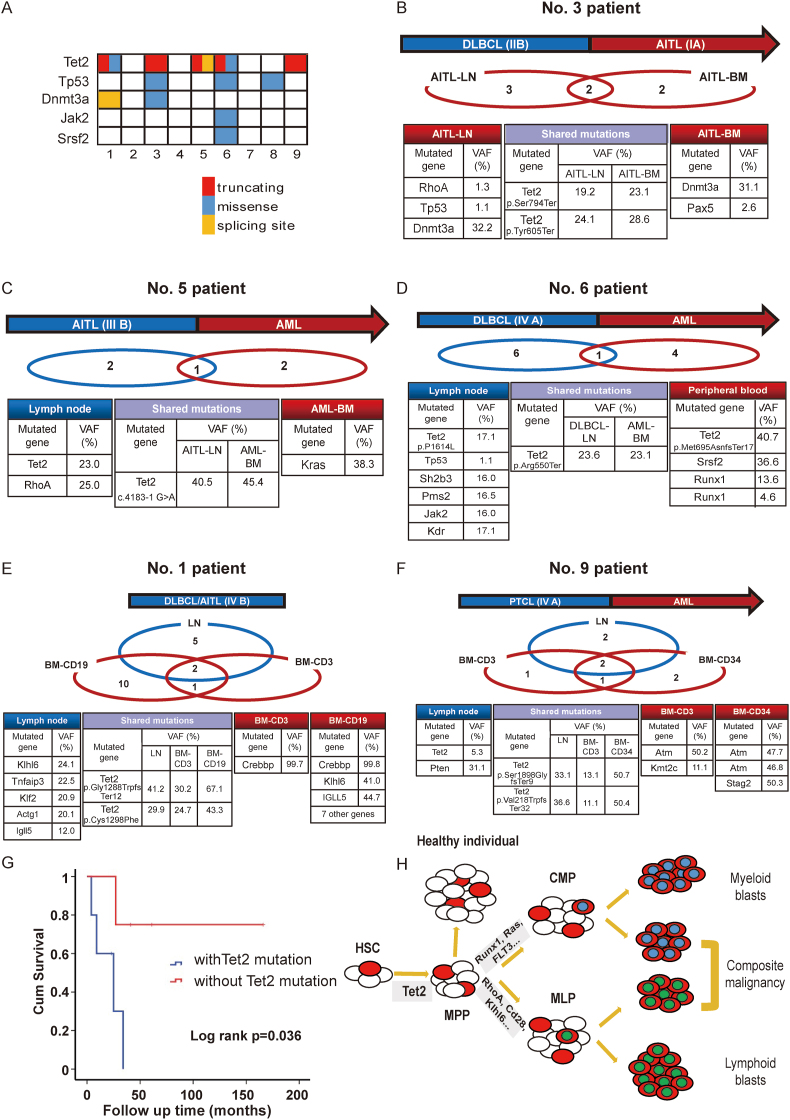
Mutation profiles of patients with Tet2 mutations and the associations with clinical outcomes. **(A)** Prevalence of CHIP-associated mutations in each patient. Each column represents a case and each row represents a gene. Different colors are used to discriminate mutations of different functional types (the red stands for truncated mutation, the blue stands for missense mutation, and the yellow stands for mutations on the splicing site). CHIP-associated gene mutations such as Tet2 (5 of 9 patients) Tp53 (3/9), Dnmt3a (2/9), Jak 2 (1/9), and Srsf2 (1/9), were highly prevalent in HCM patients. Tet2 gene mutations were exclusively detected in all the five patients, and the shared Tet2 mutations are almost truncating mutations resulting in a loss-of-function TET2 protein. **(B)** No. 3 patient was a 69 years old female patient, who was initially diagnosed with diffuse large B-cell lymphoma (DLBCL) and received standard therapy including six circles of R–CHOP (rituximab plus cyclophosphamide, doxorubicin, vincristine, and prednisone) followed by two circles of rituximab. Twenty-five months after first admission, she developed DLBCL combined with angioimmunoblastic T-cell lymphoma (AITL), in which monoclonal abnormal B cells were detected by bone marrow flow cytometry and biopsy of cervical lymph node identified the AITL diagnosis. Receptor gene rearrangement test of the lymphoid tissue indicated positive TCR (T-cell receptor) gene rearrangement and negative IGH (immunoglobulin heavy chain) gene rearrangement. Identical loss-of-function Tet2 mutations (p. Ser794Ter and p. Tyr605Ter) and one Dnmt3a mutation (p. Ala368Pro) with high variant allele frequency were both detected in the lymphoid tissue and bone marrow aspirate. Another typical RhoA mutation (p. Gly17Val) was identified in the lymphoid tissue, consistent with the diagnosis. The mutations of lymphoma tissue were marked in blue, bone marrow aspirate without evasion in angioimmunoblastic T-cell lymphoma stage was marked in red, and shared Tet2 mutations (p. Ser794Ter and p. Tyr605Ter) were marked in purple. **(C)** The No. 5 patient was 19 years old when he was diagnosed with AITL and received six circles of CHOP-like regiments. He was diagnosed with acute myeloid leukemia (AML) eight months later. Two Tet2 mutations (c. 4183-1 G > A with variant allele frequency/VAF of 40.5% and p. Leu920IlefsTer3 with VAF of 23%) and a clonal RhoA mutation (p. Gly17Val with VAF of 25%) were detected in the lymphoma cells by whole-exon sequencing. In the bone marrow aspirate in the AML stage, the same Tet2 c. 4183-1 G > A mutation (VAF 45.4%) as noted in the lymph node and another Kras p. Gly13Asp mutation (VAF 38.3%) were found, which were both absent in the germ-line controls. The mutations of the lymphoid tissue in angioimmunoblastic T-cell lymphoma stage were marked in blue, bone marrow aspirate in acute myeloid leukemia stage was marked in red, and shared Tet2 mutations (c. 4183-1 G > A) were marked in purple. **(D)** The No. 6 patient (65 years old, female) was another case with sequential onset of DLBCL and AML within 18 months. Following the diagnosis of DLBCL, the patient underwent standard therapeutic intervention, encompassing six cycles of R–CHOP followed by two cycles of rituximab. Subsequently, due to disease progression, R-MINE (rituximab plus mesna, ifosfamide, mitoxantrone, and etoposide) was administered as the second-line treatment. Then he continued treatment with venetoclax, azacytidine, and mitoxantrone after the diagnosis of AML eight months later. The lymphoid tissue in the DLBCL stage and peripheral blood in the leukemia stage shared the same initiative truncating Tet2 mutation (p. Arg550Ter) with high allele frequency, which was absent in germ-line control of oral epithelial cells. In the lymphoid tissue, a distinctive Tet2 p. Pro1614Leu mutation was identified, accompanied by mutations in Tp53, Sh2b3, Pms2, Jak2, and Kdr. Furthermore, in peripheral blood obtained at the AML stage, an additional Tet2 p. Met695AsnfsTer17 mutation was detected, along with mutations in Srsf2 and Runx 1. The mutations of the lymphoid tissue in DLBCL were marked in blue, bone marrow aspirate in acute myeloid leukemia stage was marked in red, and shared Tet2 mutations (p. Arg550Ter) were marked in purple. **(E)** The No. 1 (64 years old, male) patient was diagnosed with and double-checked as DLBCL combined with AITL (Ann Arbor stage IV with bone marrow involved), according to the morphological, pathological, immunological, and molecular characteristics of both lymph node and bone marrow aspirate. After the chemotherapy of a cycle of R–COP (rituximab plus cyclophosphamide, vincristine, and prednisone) followed by lenalidomide and VP16, the patient was discharged from the hospital upon improvement of their general condition. Subsequently, the patient was lost to follow-up. Targeted next-generation sequencing of the lymphoid tissue, sorted B-lineage cells (CD19^+^) from bone marrow aspirate, and T-lineage cells (CD3^+^) from bone marrow aspirate all identified the same Tet2 mutations (p. Gly1288TrpfsTer12 and p. Cys1298Phe). Both sorted B-lineage cells and T-lineage cells share the CREBBP p. Val1414Ile mutation. Other mutations found in the lymphoid tissue, including Klhl6, Tnfaip3, Klf2, Actg1, and Igll5, are also present in B-lineage cells. Additionally, some mutations in B-lineage cells (Fat 1, Ikzf1, Phf6, and Smarca4) are unique to this cell population. The mutations of the lymphoid tissue in DLBCL were marked in blue, sorted B-lineage cells (CD19^+^) and T-lineage cells (CD3^+^) were marked in red, and shared Tet2 mutations (p. Gly1288TrpfsTer12 and p. Cys1298Phe) were marked in purple. **(F)** The No. 9 patient (53 years old, male) was initially diagnosed with peripheral blood T-cell lymphoma, not otherwise specified, and received six circles of CHOP regiments, with the efficacy evaluation as disease progression. After the treatment of chidamide, an oral histone deacetylase inhibitor, combined with mitoxantrone, the primary disease could remain stable. Nineteen months later, the cellular morphology and flow cytometry typing results of bone marrow indicated he had developed to T-cell lymphoma combined with AML (66.5% of abnormal myeloid progenitor cells and 0.06% abnormal T cells). He received the chemotherapy of azacitidine and venetoclax for two circles, and until the last follow-up in July 2023, he was assessed as a partial remission for AML. The lymphoma tissue in lymphoma stage and sorted T-lineage (CD3^+^) cells and myeloid leukemia cells (CD34^+^) from bone marrow aspirate all identified the same Tet2 mutations (p. Ser1298GlyfsTer9 and p. Val218TrpfsTer32). In the lymphoma tissue, a distinct Tet2 p.Leu1244Pro mutation and a Pten p.Thr131Pro mutation were detected. Sorted T-lineage cells and myeloid leukemia cells both exhibited the Atm p.Cys1899X mutation, whereas a Kmt2c p.Lys339Asn mutation was specifically found in sorted T-lineage cells. Furthermore, another Atm p.Lys2749Ile mutation and the Stag 2 p.Y414X mutation were identified in sorted myeloid leukemia cells. The mutations of the lymphoid tissue in peripheral T cell lymphoma were marked in blue, sorted T-lineage cells (CD3^+^) and myeloid leukemia cells (CD34^+^) were marked in red and shared Tet2 mutations (p. Ser1298GlyfsTer9 and p. Val218TrpfsTer32) were marked in purple. **(G)** The Kaplan–Meier curve of patients stratified by Tet2 gene mutation status. The blue line represents the patients with Tet2 mutations in hematopoietic stem/progenitor cells, and the red line represents the composite hematologic malignancies without Tet2 gene mutation. The *y*-axis represents the overall survival rate, and the *x*-axis represents the follow-up time. *p* values were calculated by log-rank test. **(H)** The multi-step clone evolution model of Tet2 mutation-driven hematologic malignancies. The impairment of TET2 function caused by genetic mutation frequently appeared and could be the initiative step that discriminated the disease process of myeloid, lymphoid, or sequential onset of two hematological malignancies by cooperating with subsequent mutations or under competitive pressure. MPP, multipotential progenitor; CMP, common myeloid progenitor cell; MLP, multipotent lymphoid progenitor cells.

Further, we evaluated the association of Tet2-CHIP mutation with clinical characteristics and outcomes (Table S4). We found that extremely complex chromosome karyotypes are more likely to occur in tumor cells with Tet2 gene mutation but with no significant statistical difference. Patients possessing Tet2 gene mutations also showed a tendency towards a shorter interval between the occurrence of two malignancies (median interval time: 18 months *vs*. 34 months, *P* = 0.190) and a lower remission rate for the second tumor (40% *vs*. 75%, *P* = 0.524). Remarkedly, patients with Tet2-CHIP-associated mutations had shorter overall survival time (median: 23 months *vs*. 51 months, Log-rank *P* = 0.036) (Fig. 1G).

Herein, we systematically summarized the clinical characteristics and further analyzed the genetic background of HCM with multi-lineage involvement. We found that HCM always presents with lymphoma as the initial diagnosis. While the pathogenesis of two tumors can be stratified into two categories based on whether they have the same clonal origin. In this study, we found that five of the nine HCM patients shared a common clone origin, and Tet 2-associated mutation appeared in all HCM cases sharing common origins. Almost all the Tet2-CHIP-associated mutations were truncating mutations, which could have a damaging effect on the structure and function of the TET2 protein. Further, the subgroup of HCM patients bearing loss-of-function Tet2 mutation in HSCs/HPCs had a poor prognosis (*P* = 0.036 for lower overall survival) and were more likely to exhibit unstable chromosome, progress to a second neoplasm in a shorter time, and show poorer response to conventional therapeutics.

As is known, TET2 is a member of enzymes of the TET family (TET1, TET2, and TET3), the main biological function of which is to catalyze the oxidation of 5-methylcytosine to 5-hydroxymethylcytosine and involve DNA demethylation related gene regulation.^4^ The expression levels of different subtypes of Tet proteins vary in different tissues *in vivo*, with TET2 uniquely highly expressed in the HSCs/HPCs. It plays important roles in regulating the immune system, safeguarding genome mutagenicity, and maintaining the physiological hematopoiesis including the self-renewal of stem cells and lineage commitment. Since 2009, somatic Tet 2 mutations had been initially discovered in myeloid neoplasms, then frequently detected in various lymphomas, and proven to be commonly accumulating in CHIP. As one of the most frequent genetic alterations of CHIP, Tet2 gene mutation in HSCs/HPCs has been demonstrated to be associated with increased risk of hematologic malignancy and adverse outcome.^1^^,^^2^ Tet2 gene mutations were identified in 70%–80% of angioimmunoblastic T-cell lymphoma. Previous studies also provided evidence of the pathogenesis of lymphoma derived by Tet2 mutation in hematopoietic precursor cells.^5^ Our study provides more comprehensive evidence to illustrate that the Tet2-CHIP appeared in the early HSC/HPC stage as the initiating clone of HCM with multi-lineage involvement. The impairment of TET2 function caused by genetic mutation can be the initiative step, and subsequently collaborate with other genetic changes to promote the tumor transformation in the pathogenesis of hematologic malignancies, independently or simultaneously (Fig. 1H).

In clinical practice, more attention should be paid to these non-Hodgkin lymphomas evolved from Tet2 deficient HPCs. Close hematologic surveillance should be made and potential therapeutic strategies selectively targeting Tet2 defects of HSCs/HPCs may emerge from these studies.

## Ethics declaration

The study was approved by the ethics committee of Tongji Hospital, Tongji Medical College, Huazhong University of Science and Technology (No. TJ-IRB20220201). Informed consent was obtained by eligible patients and their families according to the Declaration of Helsinki.

## Author contributions

Jin Huang and Haolong Lin performed data analysis, wrote the paper, and contributed equally to this work. Liang Huang designed and directed the study. Xiaoxi Zhou and Xia Mao collected the clinical data and conducted the follow-up. Min Xiao made important contributions to the collation and uploading of sequence raw data. Liang Huang, Qilin Ao, and Xiaoxi Zhou meticulously reviewed and made necessary revisions to the manuscript. Qilin Ao provided support in pathological diagnosis and sample processing.

## Conflict of interests

The authors have no conflict of interests to declare.

## Funding

This work is supported by funding from the 10.13039/501100001809National Natural Science Foundation of China (No. 82070211 to Liang Huang, No. 82170167 to Xiaoxi Zhou, No. 81800356 to Jin Huang) and the National Key R&D Program of China (No. 2022YFC2502604 to Liang Huang).

## Figures and Tables

**figs1:**