# Supplementary material for: The link between ten-eleven translocation-2 (Tet2) related clonal hematopoiesis and sequential onset of two hematologic malignancies
Source: Genes Dis. 2024 Mar 22;12(1):101270. doi: 10.1016/j.gendis.2024.101270 (PMC11462244; doi:10.1016/j.gendis.2024.101270)
Supplement: Multimedia component 1 [file mmc1.doc]

| **Table S1. Clinical characteristics of 10 patients with onset of two hematological malignancies** | | | | | | | | | | | | |
| --- | --- | --- | --- | --- | --- | --- | --- | --- | --- | --- | --- | --- |
| **Patient** | **Sex** | **Age (y)** | **Illness stage** | **CR (Y/N)** | **Tissue used for NGS** | **Mutated gene profile** | **Molecular genetics / cytogenetics** | | **Interval (months)** | | **Death (Y/N)** | **OS (months)** |
| **1**^＊^ | Male | 64 | AITL+  DLBCL | N | LN | Tet2, Klhl6, Tnfaip3, Klf2, Actg1, Igll5 | Negative | / | | Y | | 4 |
|  |  |  | AITL+  DLBCL | N | BM-CD3 | Tet2, Crebbp |  |  |  |  |  |  |
|  |  |  |  |  | BM-CD19 | Tet2, Crebbp, Klhl6, Tnfaip3, Klf2, Actg1, Igll 5, Fat1, Ikzf1, Phf6, Smarca4 |  |  |  |  |  |  |
| **2** | Male | 45 | AITL | Y | NA | / | NA | 44 | | N | | 61+ |
|  |  |  | DLBCL | Y | LN | Igll 5, Tmsb4X, Kmt2d, Smarca4 |  |  | |  | |  |
| **3** | Female | 69 | DLBCL | Y | NA | / | NA | 25 | | Y | | 34 |
|  |  |  | AITL | N | BM | Tet2, Dnmt3, Pax5 | 48, XX, +3 [4] / 46, XX, [7]/ 45, XX, +8, -10, 22 [1]/ [cp]72-75, XX/X, X, +2, +3, +5, 6, 6q, +7, +8, 8p+*2, +10, t (11: ?) (q23: ?), +14, i (17q), +22, 22p+ [5] (BM) |  |  |  |  |  |
|  |  |  |  |  | LN | Tet2, Tp53, Dnmt3a, Pax5, RhoA, Smarca4, Tmem30a | TCR  rearrangement (+); IgH rearrangement  (-) (LN) |  |  |  |  |  |
| **4** | Male | 34 | cHL | Y | LN | Tnfaip3, Tnfrsf14, Ets1, Kmt2d, Pim1, Irf8, Socs1, Btg2, Cd70, Cd58, Igll5, Smarca4, Arid1a, Tbl1xr1, Prkcb | NA | 14 | | N | | 41+ |
|  |  |  | DLBCL | Y | LN | Tnfaip3, Tnfrsf14, Kmt2d, Bcl6, Bcl10, Socs1, Pim1, Btg2, Cd70, Cd58, Igll5, Smarca4, Arid1a, Tbl1xr1 | Bcl6 rearrangement (+); Bcl2 rearrangement (-); Myc rearrangement (-)(LN) |  |  |  |  |  |
| **5** | Male | 19 | AITL | Y | LN | Tet2, RhoA | 51, XY, +Y, add (2) (q37), +5, +18, +19, +21 [6]/ 46, XY [4] (LN) | 8 | | Y | | 11 |
|  |  |  | AML | N | BM | Tet2, Kras | MLL-AF9 (BM) |  |  |  |  |  |
| **6** | Female | 65 | DLBCL | Y | LN | Tet2, Tp53, Sh2b3, Pms2, Jak2, Kdr | Negative | 18 | | Y | | 25 |
|  |  |  | AML | Y | PB | Tet2, Srsf2, Runx1 | Negative |  |  |  |  |  |
| **7** | Female | 36 | cHL | Y | LN | Xpo1 | NA | 154 | | N | | 166+ |
|  |  |  | AML | Y | BM | Stag2 | Negative |  |  |  |  |  |
| **8** | Male | 56 | DLBCL | Y | LN | Tp53, Nfkbie, Socs1, Cd70, B2m, Btg1, Btg2, Tmsb4X | Bcl-6 rearrangement (LN) | 24 | | Y | | 27 |
|  |  |  | AML | Y | BM | Kras, Tyk2 | MLL-AF9 (BM) |  |  |  |  |  |
| **9** | Male | 53 | AITL | N | LN | Tet2, Pten | Negative | 19 | | N | | 23+ |
|  |  |  | AML | N | BM-CD3 | Tet2, Atm, Kmt2c |  |  |  |  |  |  |
|  |  |  |  |  | BM-CD34 | Tet2, Atm, Stag2 |  |  |  |  |  |  |
| **10** | Male | 51 | HGBL | N | / | / | Negtive | 36 | | Y | | 37 |
|  |  |  | AML | N | / | / |  |  |  |  |  |  |
| ^＊^For No. 1 patient, cervical lymph node biopsy indicated the diagnose as DLBCL combined with AITL; CR=complete remission; PR=partial remission; NGS=next-generation sequencing; | | | | | | | | | | | | |
| OS=overall survival; AITL=angioimmunoblastic T-cell lymphoma; DLBCL=diffuse large B-cell lymphoma; cHL=classical Hodgkin's lymphoma; AML=acute myeloid leukemia; LN=lymph node; | | | | | | | | | | | | |
| BM=bone marrow; PB=peripheral blood; HGBL=high grade B-cell lymphoma. | | | | | | | | | | | | |

| **Table S2. Diagnosis and therapy of 10 patients with onset of two hematological malignancies** | | | | | | | | |
| --- | --- | --- | --- | --- | --- | --- | --- | --- |
| **Patient** | **Sex** | **Age (y)** | **1st diagnosis** | **Date of 1st diagnosis** | **Therapy** | **2nd diagnosis** | **Date of 2nd diagnosis** | **Therapy** |
| **1**^＊^ | Male | 64 | AITL+DLBCL | 9/28/2020 | R-COP +Lenalidomide+VP16 | / | / | / |
| **2** | Male | 45 | AITL | 6/1/2018 | 6*CHOP +chidamide +2DA-EPORCH | DLBCL | 2/14/2022 | R+Mitoxantrone +R-PD-1 inhibitor+R-GDP |
| **3** | Female | 69 | DLBCL | 5/3/2017 | 6*R-CHOP+2*R | AITL | 6/19/2019 | chidamide+COP +Lenalidomide |
| **4** | Male | 34 | cHL | 2/11/2020 | 8*R-AVD | DLBCL | 4/12/2021 | R-ICE/RDHAP +Car-T +PD-1 inhibitor |
| **5** | Male | 19 | AITL | 11/30/2015 | 6*R/E-CHOP | AML | 8/9/2016 | Supportive treatment |
| **6** | Female | 65 | DLBCL | 12/1/2020 | 6*R-CHOP+2*R | AML | 6/25/2022 | Venetoclax +Azacytidine +Mitoxantrone |
| **7** | Female | 36 | cHL | 9/1/2009 | 8*ABVD+ASCT +ICE+6*PD-1 inhibitor | AML | 7/12/2022 | Decitabine+HAG +HD-Arac+allo-HSCT |
| **8** | Male | 56 | DLBCL | 11/27/2018 | 6*R-CHOP+2*R | AML | 2/2/2021 | Venetoclax +Azacytidine +Ara-c +PD-1 inhibitor |
| **9** | Male | 53 | AITL | 7/30/2021 | 6*CHOP+4*(Mitoxantrone+chidamide) | AML | 3/3/2023 | Venetoclax +Azacytidine |
| **10** | Male | 51 | HGBL | 9/1/2019 | 3*R-chop+R2-GVD+R-MA+CART | AML | 9/27/2022 | Decitabine+ Venetoclax |
| AITL=angioimmunoblastic T-cell lymphoma; DLBCL=diffuse large B-cell lymphoma; cHL=classical Hodgkin's lymphoma; AML=acute myeloid leukemia; | | | | | | | | |
| HGBL=high grade B-cell lymphoma; CART=chimeric antigen receptor T cells; allo-HSCT=allogeneic hematopoietic stem cell transplantation | | | | | | | | |

| **Table S3 Mutation profile of 9 patients with onset of two hematological malignancies** | | | | |
| --- | --- | --- | --- | --- |
| **Patient** | **Tissue for sequencing** | **Gene** | **Mutation** | **VAF (%)** |
| **1** | LN | Tet2 | p. Gly1288TrpfsTer12 | 41.2 |
|  |  | Tet2 | p. Cys1298Phe | 29.9 |
|  |  | Klhl6 | p. Ala93Asp | 24.1 |
|  |  | Tnfaip3 | p. Gln55SerfsTer41 | 22.5 |
|  |  | Klf2 | p. Pro246Leu | 20.9 |
|  |  | Actg1 | p. Gly13Asp | 20.1 |
|  |  | Igll5 | p. Cys31Tyr | 12.0 |
|  | BM-CD19+ | Tet2 | p. Gly1288TrpfsTer12 | 67.1 |
|  |  | Tet2 | p. Cys1298Phe | 43.3 |
|  |  | Crebbp | p. Val1414Ile | 99.8 |
|  |  | Klhl6 | p. Ala93Asp | 41.0 |
|  |  | Igll5 | p. Cys31Tyr | 44.7 |
|  |  | Tnfaip3 | p. Gln55SerfsTer41 | 41.9 |
|  |  | Actg1 | p. Gly13Asp | 51.5 |
|  |  | Fat1 | p. Leu949Val | 42.9 |
|  |  | Ikzf1 | c. *69G>A | 40.5 |
|  |  | Klf2 | p. Pro246Leu | 40.4 |
|  |  | Phf6 | p. Phe48Leu | 2.4 |
|  |  | Smarca4 | p. Leu1285Ser | 1.1 |
|  | BM-CD3+ | Tet2 | p. Gly1288TrpfsTer12 | 30.2 |
|  |  | Tet2 | p. Cys1298Phe | 24.7 |
|  |  | Crebbp | p. Val1414Ile | 99.7 |
| **2** | DLBCL-LN | Igll5 | p. Ala5Gly | 24.4 |
|  |  | Tmsb4X | p. Gln40Ter | 12.0 |
|  |  | Kmt2d | p. Gln2811ThrfsTer34 | 10.8 |
|  |  | Smarca4 | p. Gln1182Leu | 12.7 |
| **3** | AITL-BM | Tet2 | p. Ser794Ter | 23.1 |
|  |  | Tet2 | p. Tyr605Ter | 28.6 |
|  |  | Dnmt3a | p. Ala368Pro | 31.1 |
|  |  | Pax5 | p. Arg309Cys | 56.3 |
|  | AITL-LN | Tet2 | p.Tyr605Ter | 24.1 |
|  |  | Tet2 | p.Ser794Ter | 19.2 |
|  |  | Pax5 | p.Arg390Cys | 56.1 |
|  |  | Dnmt3a | p.Ala368Pro | 32.2 |
|  |  | Tp53 | p.Arg213Ter | 1.1 |
|  |  | RhoA | p.Gly17Val | 1.3 |
|  |  | Tmem30a | p.Lys166Ile | 31.9 |
|  |  | Smarca4 | p.Phe1246Ser | 2.2 |
| **4** | cHL-LN | Cd70 | p.Gly66Arg | 10.8 |
|  |  | Tnfaip3 | p.Leu286Ter | 8.3 |
|  |  | Tnfrsf14 | p.Cys96Arg | 3.9 |
|  |  | Pim1 | p.Ser78Pro | 6.5 |
|  |  | Pim1 | p.Asn7Thr | 4.2 |
|  |  | Ets1 | p.Thr10SerfsTer25 | 5.1 |
|  |  | Igll5 | p.Ser58Gly | 7.5 |
|  |  | Igll5 | p.Gly9Asp | 7.7 |
|  |  | Btg2 | p.Val17Glu | 4.9 |
|  |  | Btg2 | c.136_142+4del | 5.0 |
|  |  | Irf8 | p.Cys84Arg | 4.8 |
|  |  | Socs1 | p.Phe101Leu | 3.9 |
|  |  | Arid1a | p.Gln802SerfsTer15 | 4.3 |
|  |  | Prkcb | p.Phe20Ser | 4.1 |
|  |  | Tbl1xr1 | p.Val445Ala | 5.6 |
|  |  | Cd58 | p.Pro220_Ile226del | 6.4 |
|  |  | Cd58 | p.Ile219Val | 6.4 |
|  |  | Cd58 | p.Thr227Ala | 2.0 |
|  |  | Smarca4 | p.Arg1477Trp | 3.6 |
|  | DLBCL-LN | Tnfaip3 | p. Leu286Ter | 58.8 |
|  |  | Tnfrsf14 | p. Cys96Arg | 46.6 |
|  |  | Kmt2d | p. Arg2685Ter | 32.2 |
|  |  | Bcl6 | c. -11717G>A | 39.0 |
|  |  | Bcl6 | c. -11821T>G | 30.1 |
|  |  | Bcl10 | p. Asn1421IlefsTer6 | 18.0 |
|  |  | Socs1 | p. Phe101Leu | 31.9 |
|  |  | Pim1 | p. Ser78Pro | 26.3 |
|  |  | Btg2 | p. Ser118Pro | 25.7 |
|  |  | Btg2 | p. Val17Glu | 25.1 |
|  |  | Btg2 | p. Leu46AlafsTer60 | 22.5 |
|  |  | Cd70 | p. Gly66Arg | 48.1 |
|  |  | Cd58 | p. Ile219Val | 24.0 |
|  |  | Cd58 | p. Pro220_Ile226del | 23.8 |
|  |  | Cd58 | p. Thr227Ala | 12.0 |
|  |  | Igll5 | p. Ser58Gly | 51.2 |
|  |  | Igll5 | p. Gly9Asp | 47.9 |
|  |  | Smarca4 | p. Arg1477Trp | 29.9 |
|  |  | Arid1a | p. Gln802SerfsTer15 | 31.2 |
|  |  | Tbl1xr1 | p. Val445Ala | 30.1 |
| **5** | LN | Tet2 | c. 4183-1 G>A | 40.5 |
|  |  | Tet2 | p. L9201fsTer3 | 23.0 |
|  |  | RhoA | p. Gly17Val | 25.0 |
|  | BM | Tet2 | c. 4183-1 G>A | 45.4 |
|  |  | Kras | p. Gly13Asp | 38.3 |
| **6** | LN | Tet2 | p. Arg550Ter | 23.6 |
|  |  | Tet2 | p. Pro1614Leu | 17.1 |
|  |  | Tp53 | p. Pro151Ser | 1.1 |
|  |  | Sh2b3 | p. Pro574Ser | 16.0 |
|  |  | Pms2 | p. Asp401Tyr | 16.5 |
|  |  | Jak2 | p. His907Asn | 16.0 |
|  |  | Kdr | p. Pro839Leu | 17.1 |
|  | PB | Tet2 | p. Arg550Ter | 49.7 |
|  |  | Tet2 | p. Met695AsnfsTer17 | 40.7 |
|  |  | Srsf2 | p. Pro95Leu | 36.6 |
|  |  | Runx1 | p. Val124Asp | 13.6 |
|  |  | Runx1 | p. Gly168Glu | 4.6 |
| **7** | LN | Xpo1 | p. Glu571Lys | 2.3 |
|  | BM | Stag2 | p. Trp315Ter | 2.8 |
|  |  | Stag2 | p. Arg614Ter | 9.8 |
| **8** | LN | Tp53 | p. Val173Met | 64.5 |
|  |  | Nfkbie | p. Tyr254ArgfsTer4 | 45.4 |
|  |  | Socs1 | p. Ser143Arg | 42.9 |
|  |  | Socs1 | p. Leu177Val | 67.3 |
|  |  | Socs1 | p. Arg172Trp | 67.2 |
|  |  | Cd70 | p. Gln47Ter | 32.6 |
|  |  | B2m | p. Leu12Pro | 82.8 |
|  |  | Btg1 | p. Pro99Ser | 44.4 |
|  |  | Btg2 | p. Asn71Lys | 42.5 |
|  |  | Btg2 | p. Met74Lys | 42.5 |
|  |  | Btg2 | p. Ser158Asn | 76.8 |
|  |  | Tmsb4X | c. 100+1G>C | 93.2 |
|  |  | Tmsb4X | p. Gln37His | 42.7 |
|  | BM | Kras | p. Gly13Asp | 18.5 |
|  |  | Tyk2 | p. Tyr1080Cys | 43.9 |
| **9** | LN | Tet2 | p.Ser1898GlyfsTer9 | 33.1 |
|  |  | Tet2 | p.Val218TrpfsTer32 | 36.6 |
|  |  | Tet2 | p.Leu1244Pro | 5.3 |
|  |  | Pten | p.Thr131Pro | 31.1 |
|  | BM-CD3+ | Tet2 | p. Ser1898GlyfsTer9 | 13.1 |
|  |  | Tet2 | p.Val218TrpfsTer32 | 11.1 |
|  |  | Atm | p.Cys1899X | 50.2 |
|  |  | Kmt2c | p. Lys339Asn | 11.1 |
|  | BM-CD34+ | Tet2 | p.Ser1898GlyfsTer9 | 50.7 |
|  |  | Tet2 | p.Val218TrpfsTer32 | 50.4 |
|  |  | Atm | p.Lys2749Ile | 47.7 |
|  |  | Atm | p.Cys1899X | 46.8 |
|  |  | Stag2 | p. Y414X | 50.3 |
| LN=lymph node；BM=bone marrow; PB=peripheral blood; VAF=variant allele frequency | | | | |

| **Table S4 Clinical characteristics and outcomes between patients with and without Tet2 mutations** | | | |
| --- | --- | --- | --- |
| **Characteristics** | **patients with Tet2 mutations** | **patients without Tet2 mutations** | **p-value**^§^ |
|  | **(n=5)** | **(n=4)** |  |
| **Sex, m/f** | 3/2 | 3/1 | 1.000 |
| **Age, y** | 54±20.4 | 43±10.0 | 0.351 |
| **Ann Arbor (n, %)**^＊^ |  |  | 1.000 |
| **I-II** | (1, 20%) | (1, 25%) |  |
| **III-IV** | (4, 80%) | (3, 75%) |  |
| **Complex Karyotype (n, %)** | (2, 40%) | (0, 0%) | 0.444 |
| **CR1 (n, %)** | (3, 60%) | (4, 100%) | 0.444 |
| **CR2 (n, %)** | (2, 40%) | (3, 75%) | 0.524 |
| **Interval time (median, months)** | 18 | 34 | 0.190 |
| **Deaths (n, %)** | (4, 80%) | (1, 25%) | 0.206 |
| **Overall survival time (months)** | 23 | 51 | 0.036 |
| ^＊^evaluated according to the first diagnosis of lymphoma; CR1: complete remission rate for the first tumor; | | | |
| CR2: complete remission rate for the second primary tumor; | | | |
| ^§^p-value is compared by independent sample t test, Fisher exact test, Mann-Whitney U test, or log-rank test when appropriate. | | | |

**Fig. S1 Flow Sorting efficiency of CD3+ T-lineage cells (A) and CD19+ B-lineage (B) cells from bone marrow aspirate for NO.1 patient.**

The sorting efficiencies were evaluated by flow cytometry. Y-axis represents the sorting efficiency of T-lineage cells (99.2%) in Figure A and sorting efficiency of B-lineage cells (94.7%) in Figure B.

**The method of next-generation sequencing**

A total of 19 specimens from 9 patients were obtained, including the lymphoid tissue, bone marrow, and peripheral blood. Subsequently, bone marrow cells from patients 1 and 9 were further isolated through flow sorting to obtain the specific cell subpopulation. All samples were collected and stored properly with the informed consent of patients and their families

The sequenced panel was determined by the type of disease. 80 genes were detected in patients with AML and 157genes in patients with lymphoma. All of these genes representing the primary mutation hotspots in myeloid and lymphoid malignancies^1^. Whole exon sequencing was performed in some patients (sequencing reports were carried when admitted to our hospital). Genomic DNA isolation from the samples was performed using the QIAamp DNA Kit (Qiagen GmbH, Hilden, Germany). Following quality validation, library construction was executed using the Ion AmpliSeq Library Kit 2.0 (Applied Biosystems, Foster City, CA, USA). Quality-approved libraries were transferred to the Ion OneTouch 2 system for water-in-oil PCR to generate templates. Subsequently, next-generation sequencing was conducted on the Illumina NextSeq 500/550 platform in adherence to the established protocol. The sequencing data underwent analysis utilizing BWA, GATK, VarScan, and ANNOVAR, with references derived from the 1000 Genomes Project (1000G), gnomAD, ExAC, avsnp150, HGMD, and ClinVar database.

The available raw sequencing data have been uploaded to the SRA database

and released, retrievable through the accession number (PRJNA1067006).

1 Huang J, Zhou J, Xiao M, et al. The association of complex genetic background with the prognosis of acute leukemia with ambiguous lineage. Sci Rep. 2021 Dec 21;11(1):24290.
